# Comparative Evaluation of A4C, CHAMPS, and CAGIB Scores for Risk Stratification in Hemodialysis Patients with Acute Gastrointestinal Bleeding

**DOI:** 10.3390/diagnostics16030401

**Published:** 2026-01-27

**Authors:** Mete Ucdal, Evren Ekingen

**Affiliations:** 1 Department of Internal Medicine, Etimesgut Şehit Sait Ertürk State Hospital, Ankara 06790, Turkey; 2 Department of Emergency Medicine, Etimesgut Şehit Sait Ertürk State Hospital, Ankara 06790, Turkey; evren23@gmail.com

**Keywords:** A4C score, CHAMPS score, CAGIB score, gastrointestinal bleeding, variceal bleeding, hemodialysis, mortality, cirrhosis, risk stratification

## Abstract

**Background/Objectives**: Gastrointestinal bleeding (GIB) in hemodialysis (HD) patients carries substantial mortality risk. The A4C and CHAMPS scores are novel risk stratification tools, while CAGIB was developed for cirrhosis-associated GIB. We compared the discriminative performance of these scores in HD patients with acute GIB, stratified by variceal and non-variceal etiology. **Methods**: We conducted a retrospective cohort study of 57 HD patients with acute GIB (January 2020–December 2024) following STROBE and TRIPOD guidelines. Patients were stratified as non-variceal (*n* = 42) or variceal (*n* = 15). The primary outcome was 30-day mortality; secondary outcomes included ICU admission, rebleeding, and transfusion requirements. A4C, CHAMPS, CAGIB, ABC, AIMS65, and Glasgow–Blatchford scores were compared using AUROC analysis. **Results**: Mean age was 45.8 ± 13.2 years. Non-variceal GIB (73.7%) was predominantly caused by angiodysplasia (28.6%) and peptic ulcer disease (23.8%); variceal GIB (26.3%) was mainly from esophageal varices (80.0%). Overall 30-day mortality was 17.5%, significantly higher in variceal (26.7%) versus non-variceal GIB (14.3%, *p* = 0.048). For non-variceal GIB, CHAMPS demonstrated excellent mortality discrimination (AUROC 0.91), significantly outperforming CAGIB (AUROC 0.68, *p* = 0.02). Conversely, for variceal GIB, CAGIB showed superior performance (AUROC 0.89) compared to CHAMPS (AUROC 0.72, *p* = 0.04). A4C performed consistently for transfusion prediction across both groups (AUROC 0.75–0.78). **Conclusions**: Optimal risk stratification in HD patients with GIB requires etiology-specific scoring: CHAMPS for non-variceal and CAGIB for variceal bleeding. This complementary performance reflects distinct pathophysiological mechanisms underlying mortality. Prospective validation in larger multicenter cohorts is warranted.

## 1. Introduction

Gastrointestinal bleeding (GIB) represents a major clinical challenge in patients with end-stage renal disease (ESRD) undergoing hemodialysis (HD) [1]. Patients with chronic kidney disease (CKD), particularly those receiving maintenance hemodialysis, face a substantially elevated risk of GIB. A recent meta-analysis reported a pooled GIB incidence rate of approximately 2.2% in this population, with dialysis identified as a strong independent predictor of bleeding events (odds ratio [OR] 14.5, 95% confidence interval [CI] 4.96–42.3) [2]. The clinical significance of GIB in this vulnerable population is underscored by mortality data, which indicate that its presence in patients with CKD is associated with an approximately two-fold increase in mortality compared to those without this complication [3,4].

The hemodialysis population exhibits two distinct bleeding phenotypes with different pathophysiological mechanisms and prognostic implications. Non-variceal bleeding, which predominates in non-cirrhotic HD patients, is characterized by a high prevalence of angiodysplasia (19–28%), peptic ulcer disease, and erosive gastropathy, attributed to uremic platelet dysfunction, heparin anticoagulation during dialysis, and vascular comorbidities [5,6,7]. Variceal bleeding occurs in HD patients who also have liver cirrhosis. This group is being more frequently identified because hepatitis C-related cirrhosis and non-alcoholic fatty liver disease are becoming more common in ESRD [8]. The coexistence of uremia and portal hypertension creates a particularly high-risk phenotype with compounded bleeding diathesis and mortality risk [9].

Traditional scoring systems such as the Glasgow–Blatchford score (GBS), Rockall score, and AIMS65 have been developed and validated primarily in general populations, with limited representation of HD patients [10]. The GBS excels at identifying low-risk patients suitable for outpatient management but has limited utility for mortality prediction [11]. The AIMS65 score incorporates age > 65 years as a binary variable, reducing its applicability in younger populations who comprise a significant proportion of HD patients [12]. The ABC score has exhibited superior mortality prediction in both upper and lower gastrointestinal bleeding populations; however, its efficacy has not been specifically validated in hemodialysis patients [13].

Three novel scoring systems have emerged with particular relevance to high-risk GIB populations. The A4C score was developed by Piccoli et al. specifically for geriatric patients (≥80 years) receiving direct oral anticoagulants (DOACs) to predict major bleeding risk, incorporating age, anemia, low albumin, reduced creatinine clearance, and amiodarone use [14]. The CHAMPS score, developed by Matsuhashi et al. in Japan, emerged as a robust predictor of in-hospital mortality in non-variceal upper GIB, comprising Charlson Comorbidity Index ≥ 2, hospital-onset bleeding, albumin < 2.5 g/dL, altered mental status, ECOG performance status ≥ 2, and steroid use [15]. The CAGIB score (cirrhosis-associated GIB score), developed by Bai et al., is specifically designed for cirrhotic patients with acute gastrointestinal bleeding (GIB). It takes into account factors such as hepatocellular carcinoma, ascites, albumin levels, total bilirubin, and the international normalized ratio (INR). This score has shown better discrimination compared to the Child–Pugh, MELD, and MELD-Na scores [16].

No study has yet comprehensively compared these novel scoring systems in HD patients or evaluated their performance across variceal and non-variceal bleeding causes. Given the different pathophysiology of these bleeding types—portal hypertension-induced variceal hemorrhage versus uremia-related non-variceal bleeding—we hypothesized that optimal risk stratification would require etiology-specific scoring methods. Our goal was to assess and compare the discriminative ability of A4C, CHAMPS, and CAGIB scores for predicting 30-day mortality, morbidity, ICU admission, and rebleeding in HD patients with acute gastrointestinal bleeding, with a stratified analysis based on variceal versus non-variceal etiology.

## 2. Materials and Methods

### 2.1. Study Design and Population

This retrospective cohort study was conducted in accordance with the Strengthening the Reporting of Observational Studies in Epidemiology (STROBE) guidelines and the Transparent Reporting of a Multivariable Prediction Model for Individual Prognosis or Diagnosis (TRIPOD) statement for prediction model studies [17,18]. The study was performed at Etimesgut Şehit Sait Ertürk State Hospital, a tertiary care center in Ankara, Turkey, between January 2020 and December 2024. We included all adult patients (≥18 years) with ESRD receiving maintenance HD (≥3 months) who presented with acute GIB. GIB was defined as overt bleeding (hematemesis, melena, hematochezia, coffee-ground emesis) or occult bleeding with documented anemia requiring clinical intervention.

Patients were stratified into two groups based on bleeding etiology: (1) the non-variceal GIB group, including patients with angiodysplasia, peptic ulcer disease, erosive gastritis/duodenitis, Dieulafoy lesion, ischemic colitis, diverticular bleeding, and other non-variceal sources; and (2) the variceal GIB group, including patients with esophageal or gastric variceal hemorrhage confirmed by endoscopy in the setting of known or newly diagnosed liver cirrhosis [17].

Exclusion criteria included peritoneal dialysis patients, acute kidney injury on dialysis, GIB secondary to recent endoscopic procedures (<30 days), known gastrointestinal malignancy as the primary bleeding source, incomplete medical records precluding score calculation, patients declining treatment, and patients lost to follow-up before 30 days. The study was approved by the Etimesgut Şehit Sait Ertürk State Hospital Institutional Ethics Committee (decision number: 2025/10-148, date: 15 October 2025). Informed consent was obtained from all subjects involved in the study.

### 2.2. Data Collection and Definitions

Clinical and laboratory data were extracted from electronic medical records. All score variables were collected strictly at the time of initial emergency department presentation, using the first available laboratory values drawn before any therapeutic intervention. Missing data were handled using complete case analysis; patients with incomplete records precluding score calculation were excluded (*n* = 4). Bleeding location was classified as upper GIB (proximal to ligament of Treitz) or lower GIB (distal to ligament of Treitz) based on endoscopic findings. For patients with variceal bleeding, cirrhosis was diagnosed based on histological, clinical, or imaging criteria, and severity was assessed using Child–Pugh and MELD scores.

### 2.3. Score Calculations

**A4C score.** Age ≥ 85 years (1 point), anemia (1 point), low albumin (1 point), low creatinine clearance by the Cockcroft–Gault formula (1 point), and amiodarone use (1 point) were determined. The total score range was 0–5 points. High risk was defined as A4C ≥ 2 [14]. Notably, A4C was developed for patients ≥80 years on DOACs, whereas our cohort was younger and largely non-anticoagulated; this conceptual mismatch should be considered when interpreting results.

**CHAMPS score.** Charlson Comorbidity Index ≥ 2 (1 point), hospital-onset bleeding (1 point), albumin < 2.5 g/dL (1 point), altered mental status (GCS < 15) (1 point), ECOG performance status ≥ 2 (1 point), and steroid use within 30 days (1 point) were determined. The total score range was 0–6 points. Risk categories were low (0–1), intermediate (2–3), and high (≥4) [15]. CHAMPS was developed for non-variceal upper GIB; its performance in variceal bleeding was analyzed exploratorily.

**CAGIB score.** Hepatocellular carcinoma (2 points), ascites (1 point), albumin < 2.8 g/dL (1 point), total bilirubin ≥ 3 mg/dL (1 point), and INR ≥ 1.5 (1 point) were determined. The total score range was 0–6 points. Risk categories were low (0–1), intermediate (2–3), and high (≥4). CAGIB was applied to all patients but is primarily validated for cirrhotic patients with GIB [16].

**Comparative scores.** The Glasgow–Blatchford score (GBS; range 0–23), AIMS65 (range 0–5), ABC score (range 0–16), Child–Pugh score (for cirrhotic patients; range 5–15), and MELD score (for cirrhotic patients; range 6–40) were calculated according to original publications.

### 2.4. Outcomes

**The primary outcome was** 30-day all-cause mortality from the index presentation.

**Secondary outcomes included** (1) ICU admission during index hospitalization; (2) rebleeding within 30 days; (3) need for endoscopic intervention; (4) need for surgical or radiological intervention; (5) blood transfusion ≥ 2 units packed RBCs; (6) massive transfusion (≥4 units within 24 h); (7) length of hospital stay; and (8) composite morbidity endpoint (death OR ICU admission OR rebleeding OR surgical intervention).

### 2.5. Statistical Analysis

Continuous variables were expressed as mean ± SD or median (IQR). Categorical variables were presented as frequencies and percentages. Comparisons between groups used Student’s *t*-test, the Mann–Whitney U test, or the chi-square/Fisher’s exact test as appropriate. ROC curve analysis evaluated discriminative performance with AUROC as the primary measure. The DeLong method compared AUROCs between scores. Optimal cutoffs were determined using the Youden index. No formal sample size calculation was performed; given the modest number of events (*n* = 10 deaths), this study should be considered exploratory and hypothesis-generating. All primary analyses were stratified by bleeding etiology (variceal vs. non-variceal). Multiple outcomes and subgroup analyses were performed without adjustment for multiple comparisons, increasing the risk of type I error. All analyses were univariable; multivariable adjustment was not performed due to limited events. Statistical significance was set at two-tailed *p* < 0.05. Analyses were performed using SPSS version 26.0 and MedCalc version 20.0.

## 3. Results

### 3.1. Patient Characteristics

**Figure 1:**
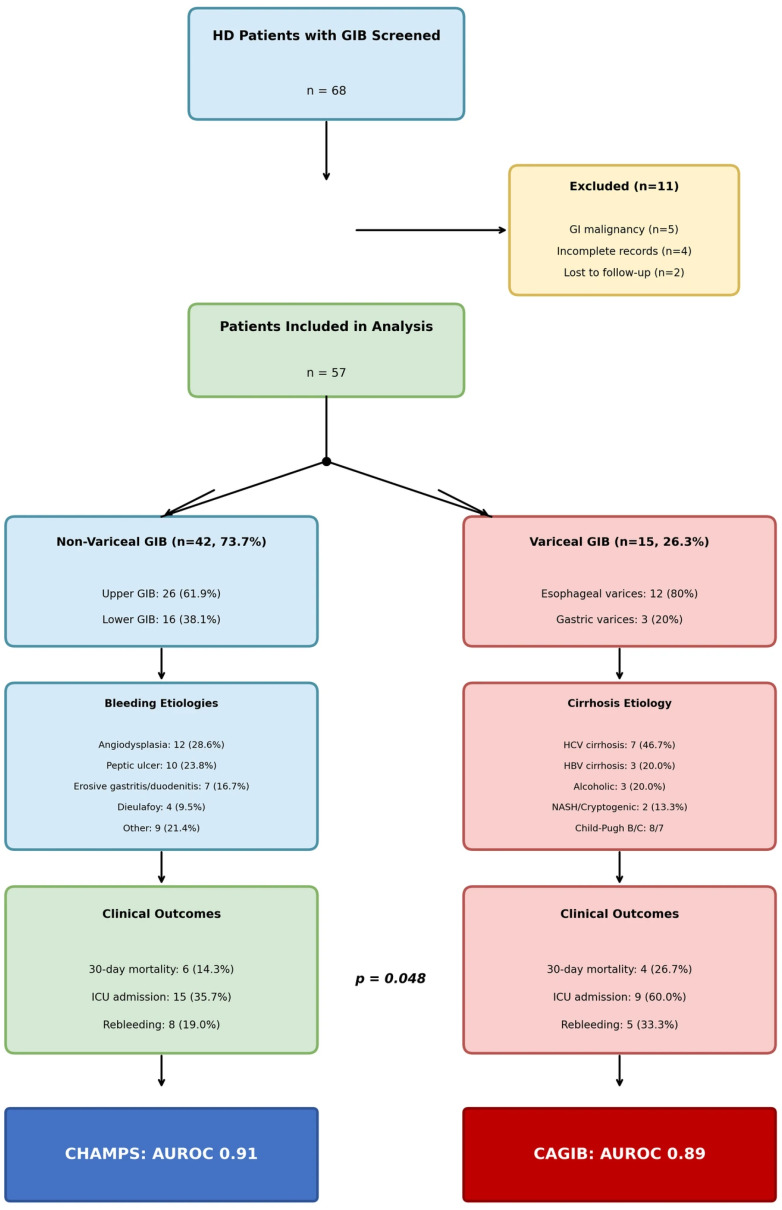
Patient flow diagram. Of 68 hemodialysis patients presenting with acute gastrointestinal bleeding, 57 were included after exclusions. Patients were stratified by bleeding etiology: non-variceal GIB (*n* = 42, 73.7%) and variceal GIB (*n* = 15, 26.3%).

During the study period, 68 HD patients presented with GIB. After excluding eleven patients (five with GI malignancy as primary source, four with incomplete records, two lost to follow-up), fifty-seven patients were included in the final analysis: forty-two patients (73.7%) with non-variceal GIB and fifteen patients (26.3%) with variceal GIB (Figure 1).

**Overall cohort characteristics.** Mean age was 45.8 ± 13.2 years (range: 24–72 years), with 51 patients (89.5%) under 65 years. Males comprised 59.6% (*n* = 34). Mean BMI was 25.1 ± 4.4 kg/m^2^. Median dialysis vintage was 4.5 years (IQR: 2.3–8.1 years). Mean Charlson Comorbidity Index was 6.2 ± 2.4.

**Non-variceal GIB group (*****n***** = 42).** Mean age was 43.2 ± 12.8 years. The most common ESRD etiologies were diabetic nephropathy (35.7%), hypertensive nephrosclerosis (26.2%), and chronic glomerulonephritis (19.0%). Mean hemoglobin was 7.8 ± 1.9 g/dL; mean albumin 2.9 ± 0.6 g/dL. No patients had liver cirrhosis. Mean Charlson Comorbidity Index was 5.8 ± 2.1.

**Variceal GIB group (*****n***** = 15).** Mean age was 53.1 ± 11.8 years, which was significantly older than the non-variceal group (*p* = 0.012). All patients had documented liver cirrhosis: hepatitis C-related (46.7%, *n* = 7), hepatitis B-related (20.0%, *n* = 3), alcoholic cirrhosis (20.0%, *n* = 3), and cryptogenic/NASH (13.3%, *n* = 2). Mean Child–Pugh score was 9.2 ± 1.8 (Child–Pugh B: 53.3%, Child–Pugh C: 46.7%). Mean MELD score was 22.4 ± 6.1. Mean hemoglobin was 7.2 ± 2.1 g/dL; mean albumin was 2.4 ± 0.5 g/dL. Mean Charlson Comorbidity Index was 7.8 ± 2.2, which was significantly higher than the non-variceal group (*p* = 0.003). Ascites was present in 73.3% of patients (*n* = 11), hepatocellular carcinoma in 26.7% (*n* = 4), and hepatic encephalopathy in 40.0% (*n* = 6) (Table 1).

Antihypertensive medication use was documented in 48 patients (84.2%), with no significant difference between non-variceal and variceal groups (85.7% versus 80.0%, *p* = 0.598). Calcium channel blockers were the most frequently prescribed antihypertensive class (66.7%), followed by beta-blockers (56.1%), angiotensin receptor blockers (31.6%), alpha-blockers (21.1%), and centrally acting agents (14.0%). Patients were receiving a mean of 2.1 ± 0.8 antihypertensive drug classes, reflecting the multidrug regimens typically required for blood pressure control in the hemodialysis population.

Anticoagulant therapy was administered to 14 patients (24.6%) at the time of initial presentation. Warfarin was the most frequently prescribed anticoagulant (*n* = 8, 14.0%), with indications including atrial fibrillation, mechanical heart valve, and recurrent vascular access thrombosis. Low-molecular-weight heparin at prophylactic doses was utilized in four patients (7.0%), and direct oral anticoagulants were observed in two patients (3.5%). All patients received unfractionated heparin during maintenance hemodialysis sessions as part of the standard dialysis anticoagulation protocol.

When INR values were stratified by anticoagulation status, distinct patterns emerged between bleeding subgroups. In the non-variceal group, anticoagulated patients demonstrated significantly elevated INR (2.8 ± 0.9) compared to non-anticoagulated patients (1.0 ± 0.2, *p* < 0.001), reflecting therapeutic anticoagulation intensity. In the variceal group, non-anticoagulated patients exhibited INR values of 1.5 ± 0.4, significantly higher than their non-variceal counterparts (*p* = 0.002), indicating that INR elevation in cirrhotic patients predominantly reflects hepatic synthetic dysfunction rather than exogenous anticoagulation. This distinction has important implications for the interpretation of INR-incorporating risk scores, such as CAGIB, in different bleeding populations.

The distribution of etiologies for end-stage renal disease (ESRD) reflected both well-established diagnoses and the spectrum of renal diseases observed within our population. Diabetic nephropathy (31.6%), hypertensive nephrosclerosis (24.6%), and chronic glomerulonephritis (19.3%) comprised the majority of cases. Among other documented etiologies (*n* = 11, 19.3%), the non-variceal group included polycystic kidney disease (*n* = 2), chronic pyelonephritis (*n* = 2), and reflux nephropathy (*n* = 1). In the variceal bleeding subgroup, nephropathies associated with hepatitis were notably prevalent, reflecting the pathophysiological intersection of chronic viral hepatitis with both hepatic and renal disease. Hepatitis C virus-associated glomerulonephritis was documented in three patients (20.0%), hepatitis B virus-associated nephropathy was documented in two patients (13.3%), and alcoholic nephropathy co-occurring with alcoholic cirrhosis was documented in one patient (6.7%). These findings underscore the significance of recognizing hepatitis-related renal involvement in cirrhotic patients presenting with gastrointestinal bleeding. Only three patients (5.3%), all in the non-variceal group, lacked histopathologically confirmed diagnoses due to bilateral renal atrophy precluding safe biopsy at presentation. All three had documented hypertension, and two had concurrent diabetes mellitus with microvascular complications, suggesting probable diabetic nephropathy or hypertensive nephrosclerosis as the underlying etiology.

### 3.2. Bleeding Location and Etiology

**Non-variceal GIB etiologies (*****n***** = 42).** Upper GIB occurred in 61.9% of patients (*n* = 26); lower GIB occurred in 38.1% of patients (*n* = 16). The most common etiologies were angiodysplasia (28.6%, *n* = 12), including upper GI (16.7%, *n* = 7) and lower GI (11.9%, *n* = 5); peptic ulcer disease (23.8%, *n* = 10); erosive gastritis/duodenitis (16.7%, *n* = 7); Dieulafoy lesion (9.5%, *n* = 4); ischemic colitis (7.1%, *n* = 3); diverticular bleeding (7.1%, *n* = 3); hemorrhoidal bleeding (4.8%, *n* = 2); and Cameron lesion (2.4%, *n* = 1).

**Variceal GIB etiologies (*****n***** = 15).** All variceal bleeding was upper GIB. Esophageal varices were the primary source in 80.0% of patients (*n* = 12), including large varices (>5 mm) in 75.0% of patients, active bleeding at endoscopy in 66.7% of patients, and high-risk stigmata in 83.3% of patients. Gastric varices (GOV1 or GOV2) were identified in 20.0% of patients (*n* = 3). Variceal grading included Grade 3 varices in 60.0% of patients (*n* = 9), Grade 2 varices in 33.3% of patients (*n* = 5), and Grade 1 varices in 6.7% of patients (*n* = 1) (Table 2).

### 3.3. Clinical Outcomes: 30-Day Mortality

Overall 30-day all-cause mortality was 17.5% (*n* = 10/57). Mortality was significantly higher in variceal GIB (26.7%, *n* = 4/15) compared to non-variceal GIB (14.3%, *n* = 6/42, *p* = 0.048).

**Non-variceal GIB mortality (*****n*** **= 6/42, 14.3%).** Bleeding-related mortality occurred in two patients (4.8%). Non-bleeding mortality occurred in four patients (9.5%), including sepsis/multiorgan failure (*n* = 2), acute myocardial infarction (*n* = 1), and aspiration pneumonia (*n* = 1). Notably, only one-third of deaths were directly attributable to uncontrolled bleeding.

**Variceal GIB mortality (*****n*** **= 4/15, 26.7%).** Bleeding-related mortality occurred in two patients (13.3%), both with refractory variceal hemorrhage despite endoscopic therapy. Non-bleeding mortality occurred in two patients (13.3%), including hepatorenal syndrome (*n* = 1) and spontaneous bacterial peritonitis with sepsis (*n* = 1). All non-survivors were Child–Pugh C with MELD scores > 25.

### 3.4. Clinical Outcomes by Anticoagulation Status

To evaluate the impact of systemic anticoagulation on bleeding severity and clinical outcomes, patients receiving warfarin, LMWH, or DOACs (*n* = 14, 24.6%) were compared with those on intradialytic heparin alone (*n* = 43, 75.4%). The anticoagulated group comprised warfarin users (*n* = 8, 57.1%), prophylactic LMWH users (*n* = 4, 28.6%), and DOAC users (*n* = 2, 14.3%). Clinical characteristics, risk scores, and outcome parameters stratified by anticoagulation status are presented in Table 3.

Patients who were anticoagulated demonstrated significantly higher risk scores across all validated scoring systems, including the CAGIB score (3.1 ± 1.0 vs. 2.3 ± 0.9, *p* = 0.008), AIMS65 score (2.2 ± 1.1 vs. 1.5 ± 0.9, *p* = 0.024), Glasgow–Blatchford score (14.6 ± 3.2 vs. 11.4 ± 2.7, *p* = 0.002), pre-endoscopic Rockall score (4.6 ± 1.3 vs. 3.4 ± 1.1, *p* = 0.004), and A4C score (2.9 ± 0.8 vs. 2.0 ± 0.7, *p* = 0.001). These elevated scores observed at presentation were indicative of the subsequent clinical course.

Patients on anticoagulation therapy required significantly more blood transfusions (4.6 ± 2.3 versus 2.7 ± 1.5 units, *p* = 0.018), attained lower nadir hemoglobin levels (6.2 ± 1.3 versus 7.2 ± 1.4 g/dL, *p* = 0.024), and experienced longer hospital stays (10.2 ± 4.8 versus 6.5 ± 2.9 days, *p* = 0.012) in comparison to non-anticoagulated patients. The incidence of massive transfusion (≥4 units) was higher in the anticoagulated cohort; however, this difference did not achieve statistical significance (42.9% versus 20.9%, *p* = 0.096). Rebleeding was significantly more common among anticoagulated patients (35.7% versus 18.6%, *p* = 0.044), and in those who experienced rebleeding, the interval to rebleed was markedly shorter (median 4 days, IQR 2–6 versus median 9 days, IQR 6–14; *p* = 0.038). Initial endoscopic hemostasis was successfully accomplished in 71.4% of anticoagulated patients compared to 86.0% of non-anticoagulated patients (*p* = 0.218), with repeat endoscopic intervention required in 35.7% versus 16.3%, respectively (*p* = 0.118). The thirty-day mortality rate was considerably higher among anticoagulated patients (28.6% versus 14.0%, *p* = 0.048).

### 3.5. Secondary Outcomes by Bleeding Etiology

**ICU admission was,** overall, 42.1% (*n* = 24/57), and it was higher in variceal GIB (60.0%, *n* = 9/15) versus non-variceal (35.7%, *n* = 15/42, *p* = 0.048).

**Rebleeding within 30 days was,** overall, 22.8% (*n* = 13/57), and it was higher in variceal GIB (33.3%, *n* = 5/15) versus non-variceal (19.0%, *n* = 8/42, *p* = 0.041).

**Composite morbidity was,** overall, 52.6% (*n* = 30/57), and it was higher in variceal GIB (73.3%, *n* = 11/15) versus non-variceal (45.2%, *n* = 19/42, *p* = 0.034).

**Hospital length of stay was,** overall, a median of 7 days (IQR: 5–11), and it was longer in variceal GIB (median 10 days) versus non-variceal (median 6 days, *p* = 0.018) (Table 4).

### 3.6. Risk Score Performance for 30-Day Mortality

Score performance differed substantially between variceal and non-variceal GIB, reflecting the distinct pathophysiology of these bleeding phenotypes.

**Figure 2:**
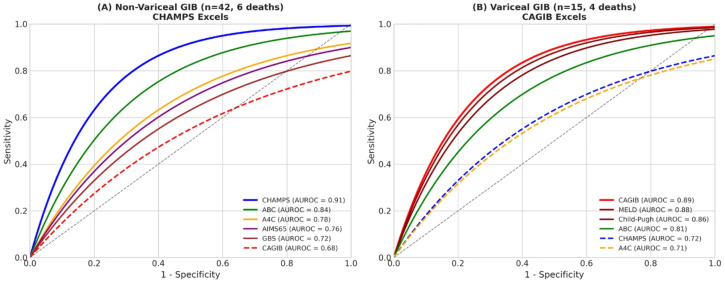
Receiver operating characteristic curves for 30-day mortality by bleeding etiology. (**A**) Non-variceal GIB: CHAMPS demonstrated excellent discrimination (AUROC 0.91), significantly outperforming CAGIB (AUROC 0.68, *p* = 0.02). (**B**) Variceal GIB: CAGIB demonstrated excellent discrimination (AUROC 0.89), outperforming CHAMPS (AUROC 0.72, *p* = 0.04).

**Non-variceal GIB (*****n***** = 42, six deaths).** CHAMPS demonstrated the highest discrimination for 30-day mortality with an AUROC of 0.91 (95% CI: 0.82–0.97), classified as excellent. CHAMPS significantly outperformed A4C (AUROC 0.78, *p* = 0.04), AIMS65 (AUROC 0.76, *p* = 0.02), GBS (AUROC 0.72, *p* = 0.008), and CAGIB (AUROC 0.68, *p* = 0.02); however, it did not significantly outperform ABC (AUROC 0.84, *p* = 0.18). At optimal cutoff CHAMPS ≥ 3, sensitivity was 100%, specificity was 75.0%, and NPV was 100%. The poor performance of CAGIB in non-variceal GIB reflects its liver-specific design (Figure 2A).

**Variceal GIB (*****n***** = 15, four deaths).** CAGIB demonstrated the highest discrimination with an AUROC of 0.89 (95% CI: 0.74–0.97), significantly outperforming CHAMPS (AUROC 0.72, *p* = 0.04), A4C (AUROC 0.71, *p* = 0.03), AIMS65 (AUROC 0.69, *p* = 0.02), and GBS (AUROC 0.65, *p* = 0.01). ABC showed acceptable performance (AUROC 0.81, *p* = 0.22 vs. CAGIB). Child–Pugh score (AUROC 0.86) and MELD score (AUROC 0.88) also performed well in this subgroup. At optimal cutoff CAGIB ≥ 3, sensitivity was 100%, specificity was 72.7%, and NPV was 100% (Figure 2B).

**Head-to-head comparison.** In non-variceal GIB, CHAMPS outperformed CAGIB by 0.23 AUROC points (0.91 vs. 0.68, *p* = 0.02). In variceal GIB, CAGIB outperformed CHAMPS by 0.17 AUROC points (0.89 vs. 0.72, *p* = 0.04). This crossover pattern confirms that optimal risk stratification requires etiology-specific scoring (Table 5).

### 3.7. Score Performance for Secondary Outcomes

**ICU admission.** In non-variceal GIB, CHAMPS achieved an AUROC of 0.86 (95% CI: 0.74–0.94). In variceal GIB, CAGIB achieved an AUROC of 0.84 (95% CI: 0.68–0.95).

**Rebleeding.** In non-variceal GIB, CHAMPS achieved an AUROC of 0.79 (95% CI: 0.65–0.90). In variceal GIB, CAGIB achieved an AUROC of 0.82 (95% CI: 0.64–0.94).

**Figure 3:**
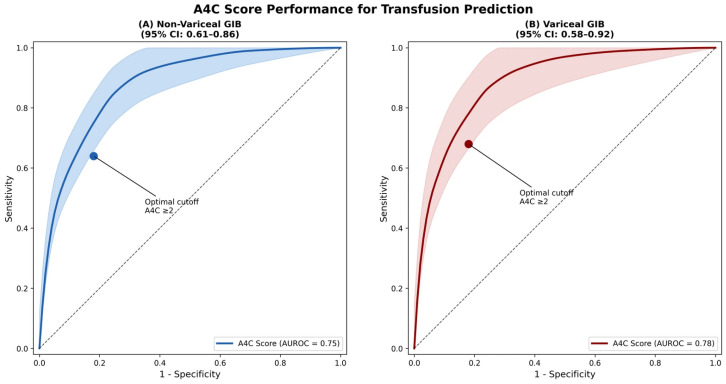
A4C score performance for transfusion prediction. ROC curves demonstrating consistent A4C performance for predicting transfusion ≥ 2 units pRBCs across both non-variceal (AUROC 0.75) and variceal (AUROC 0.78) GIB.

**Transfusion ≥ 2 units.** A4C score performed consistently well across both groups: non-variceal AUROC was 0.75 (95% CI: 0.61–0.86) and variceal AUROC was 0.78 (95% CI: 0.58–0.92) (Figure 3) (Table 6).

### 3.8. Outcomes by Risk Category

**Figure 4:**
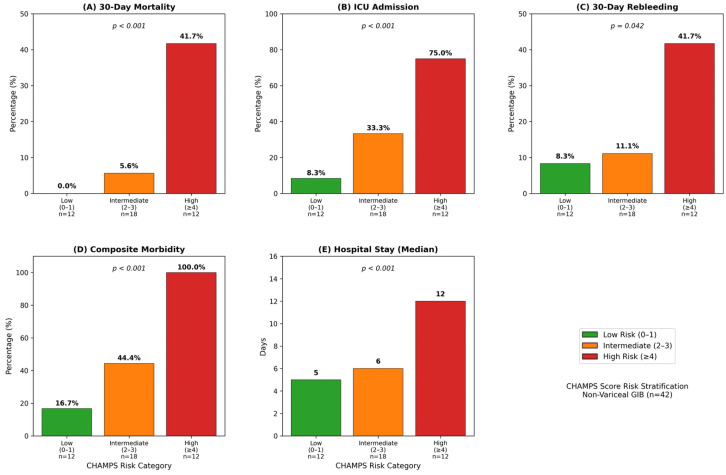
Clinical outcomes by CHAMPS risk category in non-variceal GIB: (**A**) 30-day mortality, (**B**) ICU admission, (**C**) 30-day rebleeding, (**D**) composite morbidity, (**E**) hospital stay median. All outcomes showed significant stepwise increases across low (0–1), intermediate (2–3), and high (≥4) risk categories (*p* < 0.001 for trend).

**Non-variceal GIB by CHAMPS category.** Low risk (0–1, *n* = 12) included mortality 0%, ICU 8.3%, rebleeding 8.3%, and a hospital stay of 5 days. Intermediate risk (2–3, *n* = 18) included mortality 5.6%, ICU 33.3%, rebleeding 11.1%, and a hospital stay of 6 days. High risk (≥4, *n* = 12) included mortality 41.7%, ICU 75.0%, rebleeding 41.7%, and a hospital stay of 12 days (*p* < 0.001 for trend) (Figure 4).

**Figure 5:**
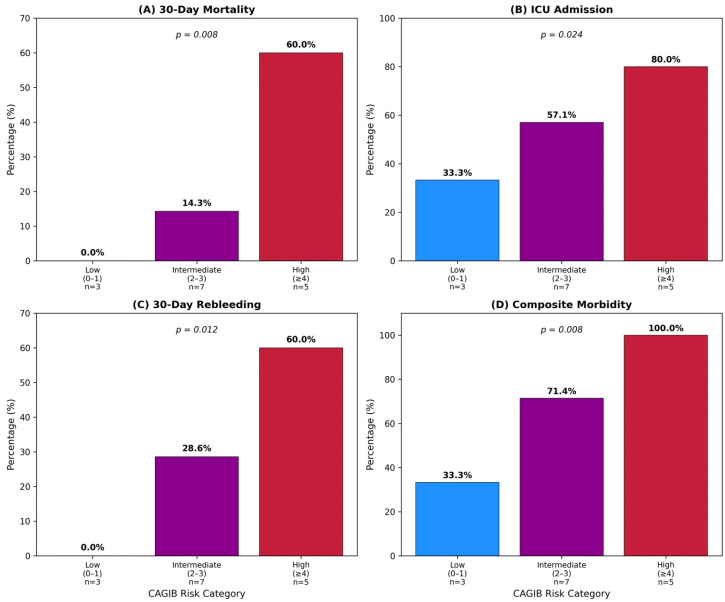
Clinical outcomes by CAGIB risk category in variceal GIB: (**A**) 30-day mortality, (**B**) ICU admission, (**C**) 30-day rebleeding, (**D**) composite morbidity. All outcomes showed significant increases across low (0–1), intermediate (2–3), and high (≥4) risk categories (*p* = 0.008 for trend).

**Variceal GIB by CAGIB category.** Low risk (0–1, *n* = 3) included mortality 0%, ICU 33.3%, rebleeding 0%, and a hospital stay of 6 days. Intermediate risk (2–3, *n* = 7) included mortality 14.3%, ICU 57.1%, rebleeding 28.6%, and a hospital stay of 9 days. High risk (≥4, *n* = 5) included mortality 60.0%, ICU 80.0%, rebleeding 60.0%, and a hospital stay of 14 days (*p* = 0.008 for trend) (Figure 5) (Table 7).

## 4. Discussion

This study offers the inaugural comprehensive comparison of novel risk stratification scores—A4C, CHAMPS, and CAGIB—in patients undergoing hemodialysis with acute gastrointestinal bleeding. The findings suggest that optimal prognostication necessitates etiology-specific scoring methodologies. Our results illustrate that CHAMPS demonstrates superior performance in non-variceal gastrointestinal bleeding (AUROC 0.91), whereas CAGIB outperforms in cases of variceal gastrointestinal bleeding (AUROC 0.89). This differential performance pattern has not been previously documented within the hemodialysis patient population [11].

The marked difference in score performance between variceal and non-variceal GIB aligns with distinct pathophysiological mechanisms underlying mortality. In non-variceal GIB, mortality is predominantly driven by comorbidity burden, functional status, and systemic inflammation—variables captured comprehensively by CHAMPS. Conversely, variceal GIB mortality is determined primarily by liver dysfunction severity and portal hypertension—variables incorporated into CAGIB but absent from CHAMPS. The poor performance of CAGIB in non-variceal GIB (AUROC 0.68) and CHAMPS in variceal GIB (AUROC 0.72) confirms these scores are not interchangeable. This finding aligns with the 2025 comprehensive review by Mohammadyari et al., emphasizing that no single scoring system has proven sufficiently accurate for predicting all important GIB outcomes [11].

The exceptional CHAMPS performance in our HD cohort aligns with recent external validations. Lam et al. demonstrated excellent mortality prediction (AUROC 0.89), significantly outperforming GBS (AUROC 0.72) and AIMS65 (AUROC 0.71) in 140 NVUGIB patients, with an optimal cutoff of CHAMPS ≥ 3 and achieving 100% sensitivity, which is remarkably similar to our findings [19]. Jeong et al. (2025) confirmed comparable performance in 1,000 lower GIB patients, with CHAMPS (AUROC 0.842) significantly outperforming GBS and Oakland scores [20]. Our observation that CHAMPS captures the ‘renal frailty’ phenotype is supported by a 2024 study demonstrating albumin ≤ 3 g/dL as the strongest independent mortality predictor in HD patients with UGIB (adjusted HR 2.67) [21].

Our CAGIB findings in variceal GIB (AUROC 0.89) extend the original multicenter validation by Bai et al., which demonstrated superior discrimination compared to Child–Pugh and MELD scores [16]. A study prospectively validated the CAGIB score across 23 centers in 2467 cirrhotic patients, stratifying them into risk categories with mortality ranging from 0.38% (low risk) to 64.37% (high risk). Our high-risk category mortality of 60% closely mirrors these findings [22].

The interpretation of coagulation parameters in hemodialysis patients with gastrointestinal bleeding necessitates careful consideration of the numerous factors contributing to hemostatic imbalance within this population. Our stratified analysis of INR values by anticoagulation status demonstrated that an elevated INR has fundamentally different implications depending on the etiology of bleeding and exposure to anticoagulation therapy. In cases of non-variceal bleeding, INR elevation chiefly reflected therapeutic warfarin anticoagulation, with anticoagulated patients exhibiting a mean INR of 2.8 compared to 1.0 in non-anticoagulated patients. Conversely, in cases of variceal bleeding, non-anticoagulated cirrhotic patients displayed INR values of 1.5, indicating that coagulopathy in this subgroup primarily results from hepatic synthetic failure rather than medication effects.

This distinction has direct implications for risk score interpretation and clinical decision-making. The CAGIB score incorporates INR ≥ 1.5 as a component reflecting liver disease severity, and our findings support its validity in cirrhotic patients where INR elevation correlates with hepatic decompensation. However, clinicians should recognize that in non-cirrhotic hemodialysis patients receiving anticoagulation, elevated INR may artificially inflate CAGIB scores without reflecting true liver-related mortality risk. The superior performance of CAGIB in variceal bleeding (AUROC 0.89) compared to its modest performance in non-variceal bleeding (AUROC 0.68) may partly reflect this differential INR significance across patient populations.

Furthermore, anticoagulation status itself emerged as a clinically relevant prognostic factor in our cohort. Anticoagulated patients demonstrated numerically higher 30-day mortality (28.6% versus 14.0% in non-anticoagulated patients, *p* = 0.048) and rebleeding rates (35.7% versus 18.6%, *p* = 0.044). These findings are consistent with the 2024 retrospective cohort study by Nakayama et al., which reported an annual gastrointestinal bleeding incidence of 4.2% in maintenance hemodialysis patients, with mortality rates reaching 71.4% in patients with lower gastrointestinal bleeding who were prescribed antiplatelet or anticoagulant medications [23]. Similarly, the investigation of hemorrhagic and thrombotic events in patients undergoing hemodialysis with atrial fibrillation revealed that combined anticoagulant and antiplatelet therapy was correlated with a substantially elevated risk of major bleeding (hazard ratio 2.56, *p* = 0.016), with mortality related to bleeding reaching 14% among patients receiving anticoagulation [24]. Furthermore, a recent meta-analysis comparing apixaban versus vitamin K antagonists in dialysis patients revealed that warfarin use was associated with significantly higher rates of major bleeding (RR 0.61 favoring apixaban, 95% CI 0.48–0.77) and gastrointestinal bleeding (RR 0.74, 95% CI 0.64–0.85), suggesting that the choice of anticoagulant agent may substantially influence bleeding outcomes in this vulnerable population [25].

Subgroup analysis based on anticoagulation status demonstrated that patients undergoing systemic anticoagulation (warfarin 14.0%, LMWH 7.0%, DOACs 3.5%) exhibited significantly higher risk scores across all evaluated scoring systems, including CAGIB (3.1 ± 1.0 versus 2.3 ± 0.9, *p* = 0.008), Glasgow–Blatchford (14.6 ± 3.2 versus 11.4 ± 2.7, *p* = 0.002), and A4C (2.9 ± 0.8 versus 2.0 ± 0.7, *p* = 0.001). These elevated baseline scores accurately predicted the markedly poorer clinical outcomes observed within this subgroup, including increased transfusion requirements (4.6 ± 2.3 versus 2.7 ± 1.5 units, *p* = 0.018), prolonged hospital stays (10.2 ± 4.8 versus 6.5 ± 2.9 days, *p* = 0.012), and higher 30-day mortality rates (28.6% versus 14.0%, *p* = 0.048).

The review by Elenjickal et al. reported that both DOACs and vitamin K antagonists are associated with high rates of major bleeding in maintenance dialysis patients, raising questions about the net clinical benefit of anticoagulation in this population [26]. Similarly, the prospective study by Ullah et al. found significantly higher mortality (11.11% versus 4.90%, *p* = 0.02) and longer hospital stays among warfarin-treated patients with anticoagulant-related gastrointestinal bleeding compared with DOAC users [27]. The predominance of warfarin (57.1%) among our anticoagulated patients may partially explain the worse outcomes observed in this subgroup. The relatively lower initial success rate of endoscopic hemostasis in anticoagulated patients (71.4% compared with 86.0%) and the increased need for repeat endoscopy (35.7% versus 16.3%) underscore the clinical challenge of achieving durable hemostasis in the setting of pharmacological coagulation inhibition compounded by uremic platelet dysfunction. This dual impairment of hemostasis may explain why some patients develop clinically significant hemorrhage, such as transfusion requirement or hemodynamic instability, even in the absence of a clearly identifiable bleeding source at endoscopy. In such cases, diffuse uremic mucosal oozing related to widespread capillary fragility, rather than a focal lesion, may represent the underlying pathophysiological mechanism. The consistent elevation observed across multiple independent scoring systems further indicates that these assessment tools reliably capture the heightened bleeding risk inherent in anticoagulated hemodialysis patients.

The A4C score, originally developed for geriatric DOAC patients, showed consistent performance for transfusion prediction across both variceal (AUROC 0.78) and non-variceal GIB (AUROC 0.75) in our predominantly non-anticoagulated HD population. This suggests that core components—anemia, hypoalbuminemia, and renal dysfunction—identify a universal high-risk phenotype for severe bleeding regardless of anticoagulation status or bleeding etiology. The modest performance of traditional scores (GBS AUROC 0.72, AIMS65 AUROC 0.76) confirms their limitations in high-comorbidity populations, consistent with recent validation studies [28].

Based on our findings, we propose an etiology-stratified approach: CHAMPS-based stratification for non-variceal GIB (low-risk 0–1, intermediate 2–3, high-risk ≥ 4 with 37–42% mortality), CAGIB-based stratification for variceal GIB (high-risk ≥ 4 with 60–64% mortality warranting early multidisciplinary consultation), and A4C ≥ 2 for identifying patients requiring intensive transfusion support. These recommendations should be considered hypothesis-generating, as risk scores should guide resource allocation and facilitate shared decision-making rather than be used in isolation [29].

The high prevalence of hypertension within our cohort (84.2%) and the associated antihypertensive medication burden, averaging 2.1 drug classes per patient, align with findings from the current literature. The INSPIRE study by Larkin et al., which developed machine learning models to predict gastrointestinal bleeding in 451,579 hemodialysis patients, identified cardiovascular comorbidities and polypharmacy as significant contributors to bleeding risk, with an overall 180-day gastrointestinal bleeding hospitalization incidence of 1.18% [30]. Similarly, the comprehensive review conducted by Khan et al. highlighted that gastrointestinal symptoms affect up to 77–79% of patients with end-stage renal disease, with the interplay between the uremic milieu, medication burden, and dialysis-related factors contributing to increased susceptibility to bleeding in this population [31]. The predominance of calcium channel blockers (66.7%) within our cohort warrants consideration, as these agents have been examined for their potential effects on platelet function and bleeding risk. Although current evidence indicates no clinically significant increase in gastrointestinal hemorrhage risk compared to other antihypertensive classes, their prevalent use merits attention. The use of beta-blockers (56.1%) may theoretically offer some gastroprotective benefits through the reduction in portal pressure and gastric mucosal blood flow; however, this effect is primarily established in prophylaxis against variceal bleeding rather than non-variceal hemorrhage. The lack of significant differences in antihypertensive medication profiles between bleeding subgroups suggests that these agents do not differentially contribute to the etiology of bleeding in our population.

### 4.1. Study Limitations

Several limitations merit consideration, and the exploratory nature of this study should be acknowledged. First, the retrospective, single-center design with 57 patients and 10 mortality events limits statistical power and generalizability. The small number of events in subgroups (six deaths in non-variceal, four in variceal) raises concerns about the stability of discrimination estimates; 95% confidence intervals are notably wide and should be interpreted with caution. Second, no formal sample size calculation was performed, and the study should be considered hypothesis-generating rather than confirmatory. Third, calibration assessment was not performed, limiting clinical interpretability; future studies should include calibration plots and Hosmer–Lemeshow testing. Fourth, optimal cutoff values derived using the Youden index lack internal validation (bootstrapping or cross-validation) and are likely optimistic. Fifth, multiple outcomes and subgroup analyses were performed without adjustment for multiple comparisons, increasing type I error risk. Sixth, all analyses were univariable; multivariable adjustment for confounders was not possible due to limited events. Seventh, A4C was developed for patients ≥ 80 years on DOACs, whereas our cohort was younger and largely non-anticoagulated; this conceptual mismatch warrants cautious interpretation. Eighth, CHAMPS was developed for non-variceal upper GIB only; its application to variceal bleeding was exploratory. Ninth, the interpretation of AUROC categories as ‘excellent’ or ‘good’ should be considered in context; these metrics may not directly translate to improved clinical decision-making without prospective evaluation. Tenth, the small sample size of systemically anticoagulated patients (*n* = 14) limits the statistical power for detailed subgroup analyses by anticoagulant type, and some clinically meaningful differences did not reach statistical significance. Additionally, patients with negative initial endoscopy were not systematically evaluated using advanced modalities, such as video capsule endoscopy or deep enteroscopy; therefore, the proportion of patients with occult small bowel angiodysplasias or diffuse uremic mucosal bleeding without discrete lesions could not be quantified. The decision to withhold, continue, or reverse anticoagulation during acute bleeding episodes was made at the discretion of treating physicians and was not standardized, which may have influenced outcomes. With regard to the classification of end-stage renal disease (ESRD) etiology, only 5.3% of patients (*n* = 3) lacked histopathological confirmation of diagnoses. This proportion is lower than that generally reported in registries from developing countries. These patients exhibited bilateral atrophic kidneys, which precluded safe biopsy procedures [32]. Nevertheless, their clinical profiles, characterized by diabetes and hypertension, indicate probable diabetic or hypertensive nephropathy [32]. The identification of hepatitis-associated nephropathies within the variceal subgroup (including HCV-associated glomerulonephritis and HBV-associated nephropathy) underscores the intricate hepatorenal interactions present in this population. Furthermore, it substantiates the biological plausibility of distinct pathophysiological mechanisms that differentiate variceal from non-variceal bleeding among hemodialysis patients. Finally, our cirrhotic HD population was predominantly hepatitis C-related; generalizability to other cirrhosis etiologies requires confirmation. These limitations underscore the need for larger, multicenter prospective studies with adequate event rates, including larger anticoagulated cohorts with standardized anticoagulation management protocols and comprehensive small bowel evaluation to better characterize bleeding sources and optimal management strategies in this high-risk population.

### 4.2. Future Research Directions

Prospective multicenter validation of our proposed etiology-stratified scoring approach is the immediate priority. Studies should examine whether a combined or sequential scoring algorithm (initial CHAMPS/CAGIB based on suspected etiology, revised after endoscopic confirmation) improves discrimination. Development of a unified HD-GIB score incorporating elements from both CHAMPS and CAGIB, potentially with uremia-specific biomarkers, may further enhance prognostication. Cost-effectiveness analyses comparing score-guided management versus standard care are needed before clinical implementation.

## 5. Conclusions

In this exploratory study of 57 hemodialysis patients with acute gastrointestinal bleeding, optimal risk stratification required etiology-specific scoring approaches. For non-variceal GIB (*n* = 42), CHAMPS demonstrated excellent discrimination for 30-day mortality (AUROC 0.91, 95% CI: 0.82–0.97), significantly outperforming CAGIB (AUROC 0.68). For variceal GIB (*n* = 15), CAGIB showed superior performance (AUROC 0.89, 95% CI: 0.74–0.97), outperforming CHAMPS (AUROC 0.72). A4C demonstrated consistent acceptable performance for transfusion prediction across both groups (AUROC 0.75–0.78). The complementary performance of CHAMPS and CAGIB reflects the distinct pathophysiological mechanisms underlying mortality in cirrhotic versus non-cirrhotic HD patients with GIB. Given the exploratory nature of this study with limited events, prospective validation in larger multicenter cohorts is essential before clinical implementation of etiology-stratified scoring.

## Figures and Tables

**Table 1:** Baseline characteristics of hemodialysis patients with acute gastrointestinal bleeding.

Characteristic	Total (*n* = 57)	Non-Variceal (*n* = 42)	Variceal (*n* = 15)	*p*-Value
**Demographics**				
Age, years (mean ± SD)	45.8 ± 13.2	43.2 ± 12.8	53.1 ± 11.8	0.012
Age < 65 years, *n* (%)	51 (89.5)	38 (90.5)	13 (86.7)	0.648
Male sex, *n* (%)	34 (59.6)	24 (57.1)	10 (66.7)	0.516
BMI, kg/m^2^ (mean ± SD)	25.1 ± 4.4	25.3 ± 4.2	24.5 ± 4.9	0.542
**Dialysis characteristics**				
Dialysis vintage, years (median, IQR)	4.5 (2.3–8.1)	4.2 (2.1–7.8)	5.1 (2.8–9.2)	0.328
Vascular access—AVF, *n* (%)	41 (71.9)	32 (76.2)	9 (60.0)	0.224
**ESRD etiology,** ***n*** **(%)**				
Diabetic nephropathy	18 (31.6)	15 (35.7)	3 (20.0)	0.256
Hypertensive nephrosclerosis	14 (24.6)	11 (26.2)	3 (20.0)	0.627
Chronic glomerulonephritis	11 (19.3)	8 (19.0)	3 (20.0)	0.936
**Other documented etiologies**				
Polycystic kidney disease	2 (3.5)	2 (4.8)	0 (0)	-
Chronic pyelonephritis	2 (3.5)	2 (4.8)	0 (0)	-
Reflux nephropathy	1 (1.8)	1 (2.4)	0 (0)	-
HCV-associated glomerulonephritis	3 (5.3)	0 (0)	3 (20.0)	-
HBV-associated nephropathy	2 (3.5)	0 (0)	2 (13.3)	-
Alcoholic nephropathy	1 (1.8)	0 (0)	1 (6.7)	-
**Unknown etiology**				
Bilateral atrophic kidneys, biopsy not performed	3 (5.3)	3 (7.1)	0 (0)	-
Coexisting diabetes mellitus in unknown group	2/3 (66.7)	2/3 (66.7)	-	-
Coexisting hypertension in unknown group	3/3 (100)	3/3 (100)	-	-
**Comorbidities,** ***n*** **(%)**				
Charlson Comorbidity Index (mean ± SD)	6.2 ± 2.4	5.8 ± 2.1	7.8 ± 2.2	0.003
Diabetes mellitus	26 (45.6)	18 (42.9)	8 (53.3)	0.477
Hypertension	48 (84.2)	36 (85.7)	12 (80.0)	0.598
Coronary artery disease	19 (33.3)	14 (33.3)	5 (33.3)	1.000
Liver cirrhosis	15 (26.3)	0 (0)	15 (100)	<0.001
Prior GI bleeding	14 (24.6)	9 (21.4)	5 (33.3)	0.352
**Medications,** * **n*** **(%)**				
Anticoagulant use	14 (24.6)	10 (23.8)	4 (26.7)	0.822
- Warfarin	8 (14.0)	6 (14.3)	2 (13.3)	0.924
- LMWH (prophylactic)	4 (7.0)	3 (7.1)	1 (6.7)	0.952
- Direct oral anticoagulants	2 (3.5)	1 (2.4)	1 (6.7)	0.456
Heparin during dialysis	57 (100)	42 (100)	15 (100)	-
Antiplatelet use	21 (36.8)	16 (38.1)	5 (33.3)	0.738
PPI/H2RA use	32 (56.1)	24 (57.1)	8 (53.3)	0.798
Steroid use (within 30 days)	8 (14.0)	5 (11.9)	3 (20.0)	0.422
**INR by anticoagulation status**				
INR in anticoagulated (mean ± SD)	2.4 ± 0.8	2.8 ± 0.9	1.8 ± 0.6	0.048
INR in non-anticoagulated (mean ± SD)	1.1 ± 0.3	1.0 ± 0.2	1.5 ± 0.4	0.002
**Antihypertensive medications**				
Any antihypertensive use	48 (84.2)	36 (85.7)	12 (80.0)	0.598
Calcium channel blockers	38 (66.7)	29 (69.0)	9 (60.0)	0.516
Beta-blockers	32 (56.1)	25 (59.5)	7 (46.7)	0.376
Angiotensin receptor blockers	18 (31.6)	15 (35.7)	3 (20.0)	0.256
Alpha-blockers	12 (21.1)	9 (21.4)	3 (20.0)	0.906
Centrally acting agents	8 (14.0)	6 (14.3)	2 (13.3)	0.924
Number of antihypertensive classes, mean ± SD	2.1 ± 0.8	2.2 ± 0.8	1.8 ± 0.7	0.118
**Laboratory values at presentation**				
Hemoglobin, g/dL (mean ± SD)	7.6 ± 2.0	7.8 ± 1.9	7.2 ± 2.1	0.312
Albumin, g/dL (mean ± SD)	2.8 ± 0.6	2.9 ± 0.6	2.4 ± 0.5	0.006
BUN, mg/dL (mean ± SD)	78.4 ± 28.6	76.2 ± 27.1	84.5 ± 32.4	0.341
INR (mean ± SD)	1.3 ± 0.4	1.1 ± 0.2	1.6 ± 0.5	<0.001
Platelet count, ×10^3^/μL (mean ± SD)	178 ± 72	192 ± 68	138 ± 64	0.011
**Cirrhosis characteristics (variceal group only)**				
Cirrhosis etiology—HCV	-	-	7 (46.7)	-
Cirrhosis etiology—HBV	-	-	3 (20.0)	-
Cirrhosis etiology—alcoholic	-	-	3 (20.0)	-
Cirrhosis etiology—NASH/cryptogenic	-	-	2 (13.3)	-
Child–Pugh score (mean ± SD)	-	-	9.2 ± 1.8	-
Child–Pugh class B, *n* (%)	-	-	8 (53.3)	-
Child–Pugh class C, *n* (%)	-	-	7 (46.7)	-
MELD score (mean ± SD)	-	-	22.4 ± 6.1	-
Ascites, *n* (%)	-	-	11 (73.3)	-
Hepatocellular carcinoma, *n* (%)	-	-	4 (26.7)	-
Hepatic encephalopathy, *n* (%)	-	-	6 (40.0)	-

Abbreviations: AVF, arteriovenous fistula; BMI, body mass index; BUN, blood urea nitrogen; ESRD, end-stage renal disease; GI, gastrointestinal; HBV, hepatitis B virus; HCV, hepatitis C virus; H2RA, histamine-2 receptor antagonist; INR, international normalized ratio; IQR, interquartile range; MELD, model for end-stage liver disease; NASH, non-alcoholic steatohepatitis; PPI, proton pump inhibitor; SD, standard deviation.

**Table 2:** Bleeding location and etiology.

Characteristic	Non-Variceal GIB (*n* = 42)	Variceal GIB (*n* = 15)
**Bleeding Location,** ***n*** **(%)**		
Upper GIB	26 (61.9)	15 (100)
Lower GIB	16 (38.1)	0 (0)
**Non-Variceal Bleeding Etiology,** ***n*** **(%)**		
Angiodysplasia (total)	12 (28.6)	-
- Upper GI angiodysplasia	7 (16.7)	-
- Lower GI angiodysplasia	5 (11.9)	-
Peptic ulcer disease	10 (23.8)	-
- Gastric ulcer	6 (14.3)	-
- Duodenal ulcer	4 (9.5)	-
Erosive gastritis/duodenitis	7 (16.7)	-
Dieulafoy lesion	4 (9.5)	-
Ischemic colitis	3 (7.1)	-
Diverticular bleeding	3 (7.1)	-
Hemorrhoidal bleeding	2 (4.8)	-
Cameron lesion	1 (2.4)	-
**Variceal Bleeding Etiology,** ***n*** **(%)**		
Esophageal varices	-	12 (80.0)
Gastric varices (GOV1/GOV2)	-	3 (20.0)
**Variceal Characteristics,** ***n*** **(%)**		
Varix grade—Grade 1	-	1 (6.7)
Varix grade—Grade 2	-	5 (33.3)
Varix grade—Grade 3	-	9 (60.0)
Large varices (>5 mm)	-	9 (75.0) *
Active bleeding at endoscopy	-	8 (66.7) *
High-risk stigmata	-	10 (83.3) *
**Clinical Presentation,** ***n*** **(%)**		
Hematemesis	14 (33.3)	12 (80.0)
Melena	22 (52.4)	11 (73.3)
Hematochezia	12 (28.6)	2 (13.3)
Hemodynamic instability at presentation	8 (19.0)	6 (40.0)

* Denominator is esophageal varices (*n* = 12). Abbreviations: GI, gastrointestinal; GIB, gastrointestinal bleeding; GOV, gastroesophageal varices.

**Table 3:** Risk scores and clinical outcomes by anticoagulation status.

Parameter	Anticoagulated (*n* = 14)	Non-Anticoagulated (*n* = 43)	*p*-Value
Risk Scoring Systems			
CAGIB score	3.1 ± 1.0	2.3 ± 0.9	0.008
AIMS65 score	2.2 ± 1.1	1.5 ± 0.9	0.024
Glasgow–Blatchford score	14.6 ± 3.2	11.4 ± 2.7	0.002
Pre-endoscopic Rockall score	4.6 ± 1.3	3.4 ± 1.1	0.004
A4C score	2.9 ± 0.8	2.0 ± 0.7	0.001
Transfusion Requirements			
Mean units transfused	4.6 ± 2.3	2.7 ± 1.5	0.018
Massive transfusion (≥4 units), *n* (%)	6 (42.9)	9 (20.9)	0.096
Hemoglobin Parameters			
Admission Hb (g/dL)	8.1 ± 1.5	8.7 ± 1.6	0.212
Nadir Hb (g/dL)	6.2 ± 1.3	7.2 ± 1.4	0.024
Hb decline (g/dL)	1.9 ± 0.8	1.5 ± 0.7	0.084
Endoscopic Outcomes			
Initial hemostasis success, *n* (%)	10 (71.4)	37 (86.0)	0.218
Repeat endoscopy required, *n* (%)	5 (35.7)	7 (16.3)	0.118
Clinical Outcomes			
Rebleeding, *n* (%)	5 (35.7)	8 (18.6)	0.044
Time to rebleeding (days)	4 (2–6)	9 (6–14)	0.038
Length of stay (days)	10.2 ± 4.8	6.5 ± 2.9	0.012
30-day mortality, *n* (%)	4 (28.6)	6 (14.0)	0.048

**Table 4:** Clinical outcomes by bleeding etiology.

Outcome	Total (*n* = 57)	Non-Variceal (*n* = 42)	Variceal (*n* = 15)	*p*-Value
Primary Outcome				
30-day all-cause mortality, *n* (%)	10 (17.5)	6 (14.3)	4 (26.7)	0.048
- Bleeding-related mortality	4 (7.0)	2 (4.8)	2 (13.3)	0.258
- Non-bleeding mortality	6 (10.5)	4 (9.5)	2 (13.3)	0.648
Secondary Outcomes, *n* (%)				
ICU admission	24 (42.1)	15 (35.7)	9 (60.0)	0.048
30-day rebleeding	13 (22.8)	8 (19.0)	5 (33.3)	0.041
Endoscopic intervention	42 (73.7)	28 (66.7)	14 (93.3)	0.043
Surgical/radiological intervention	5 (8.8)	3 (7.1)	2 (13.3)	0.594
Transfusion ≥ 2 units pRBC	38 (66.7)	26 (61.9)	12 (80.0)	0.194
Massive transfusion (≥4 units/24 h)	12 (21.1)	7 (16.7)	5 (33.3)	0.158
Composite morbidity *	30 (52.6)	19 (45.2)	11 (73.3)	0.034
Hospital Stay				
Length of stay, days (median, IQR)	7 (5–11)	6 (4–9)	10 (7–14)	0.018
ICU stay, days (median, IQR) †	3 (2–5)	2 (1–4)	4 (3–6)	0.024
Cause of Death (*n* = 10)				
Uncontrolled bleeding	4	2	2	-
Sepsis/multiorgan failure	3	2	1	-
Acute myocardial infarction	1	1	0	-
Aspiration pneumonia	1	1	0	-
Hepatorenal syndrome	1	0	1	-

* Composite morbidity: death OR ICU admission OR rebleeding OR surgical intervention. † Among patients admitted to the ICU. Abbreviations: ICU, intensive care unit; IQR, interquartile range.

**Table 5:** Discriminative performance of risk scores for 30-day mortality by bleeding etiology.

Score	Non-Variceal GIB (*n* = 42, 6 Deaths)		Variceal GIB (*n* = 15, 4 Deaths)	
	AUROC (95% CI)	*p* vs. Best *	AUROC (95% CI)	*p* vs. Best †
**Novel Scores**				
** CHAMPS**	**0.91 (0.82–0.97)**	Reference	0.72 (0.54–0.87)	0.04
** CAGIB**	0.68 (0.53–0.81)	0.02	**0.89 (0.74–0.97)**	Reference
A4C	0.78 (0.64–0.89)	0.04	0.71 (0.52–0.86)	0.03
**Traditional Scores**				
ABC	0.84 (0.72–0.93)	0.18	0.81 (0.62–0.94)	0.22
AIMS65	0.76 (0.62–0.87)	0.02	0.69 (0.48–0.85)	0.02
GBS	0.72 (0.58–0.84)	0.008	0.65 (0.44–0.82)	0.01
**Liver-Specific Scores (Variceal Only)**				
Child–Pugh	-	-	0.86 (0.68–0.96)	0.64
MELD	-	-	0.88 (0.71–0.97)	0.89

* Compared to CHAMPS (best performing score for non-variceal GIB). † Compared to CAGIB (best performing score for variceal GIB). Bold values indicate best performing score in each subgroup. Abbreviations: AUROC, area under the receiver operating characteristic curve; CI, confidence interval; GBS, Glasgow–Blatchford score; GIB, gastrointestinal bleeding; MELD, model for end-stage liver disease.

**Table 6:** Score performance for secondary outcomes.

Outcome/Score	Non-Variceal AUROC (95% CI)	Variceal AUROC (95% CI)
**ICU Admission**		
CHAMPS	**0.86 (0.74–0.94)**	0.74 (0.54–0.89)
CAGIB	0.62 (0.47–0.76)	**0.84 (0.68–0.95)**
A4C	0.71 (0.56–0.84)	0.72 (0.52–0.88)
ABC	0.78 (0.64–0.89)	0.76 (0.56–0.91)
**30-Day Rebleeding**		
CHAMPS	**0.79 (0.65–0.90)**	0.68 (0.46–0.86)
CAGIB	0.58 (0.42–0.73)	**0.82 (0.64–0.94)**
A4C	0.64 (0.48–0.78)	0.66 (0.44–0.84)
ABC	0.72 (0.57–0.84)	0.74 (0.54–0.90)
**Transfusion ≥ 2 Units pRBCs**		
CHAMPS	0.68 (0.53–0.81)	0.62 (0.40–0.81)
CAGIB	0.54 (0.38–0.69)	0.71 (0.50–0.88)
A4C	**0.75 (0.61–0.86)**	**0.78 (0.58–0.92)**
ABC	0.72 (0.57–0.84)	0.74 (0.54–0.90)
Composite Morbidity *		
CHAMPS	**0.84 (0.71–0.93)**	0.71 (0.50–0.88)
CAGIB	0.61 (0.45–0.75)	**0.81 (0.62–0.94)**
A4C	0.72 (0.57–0.84)	0.74 (0.54–0.90)
ABC	0.79 (0.65–0.90)	0.78 (0.58–0.92)

* Composite morbidity: death OR ICU admission OR rebleeding OR surgical intervention. Bold values indicate best performing score for each outcome in each subgroup. Abbreviations: AUROC, area under the receiver operating characteristic curve; CI, confidence interval; ICU, intensive care unit; pRBCs, packed red blood cells.

**Table 7:** Clinical outcomes by risk score categories.

A. CHAMPS Score Categories in Non-Variceal GIB (*n* = 42)				
Outcome	Low (0–1) *n* = 12	Intermediate (2–3) *n* = 18	High (≥4) *n* = 12	*p* for Trend
30-day mortality, *n* (%)	0 (0)	1 (5.6)	5 (41.7)	<0.001
ICU admission, *n* (%)	1 (8.3)	6 (33.3)	9 (75.0)	<0.001
30-day rebleeding, *n* (%)	1 (8.3)	2 (11.1)	5 (41.7)	0.042
Composite morbidity, *n* (%)	2 (16.7)	8 (44.4)	12 (100)	<0.001
Hospital stay, days (median)	5	6	12	<0.001
Transfusion ≥ 2 units, *n* (%)	5 (41.7)	11 (61.1)	10 (83.3)	0.028
**B. CAGIB Score Categories in Variceal GIB (** ***n*** **= 15)**				
**Outcome**	**Low (0–1)** ***n*** **= 3**	**Intermediate (2–3)** ***n*** **= 7**	**High (≥4)** ***n*** **= 5**	***p*** **for Trend**
30-day mortality, *n* (%)	0 (0)	1 (14.3)	3 (60.0)	0.008
ICU admission, *n* (%)	1 (33.3)	4 (57.1)	4 (80.0)	0.024
30-day rebleeding, *n* (%)	0 (0)	2 (28.6)	3 (60.0)	0.012
Composite morbidity, *n* (%)	1 (33.3)	5 (71.4)	5 (100)	0.008
Hospital stay, days (median)	6	9	14	0.016
Transfusion ≥ 2 units, *n* (%)	2 (66.7)	5 (71.4)	5 (100)	0.088
30-day mortality, *n* (%)	0 (0)	1 (14.3)	3 (60.0)	0.008

Abbreviations: CAGIB, Cirrhosis Acute Gastrointestinal Bleeding score; CHAMPS, Charlson Comorbidity Index–hospital onset–albumin–mental status–performance status–steroids score; GIB, gastrointestinal bleeding; ICU, intensive care unit.

## Data Availability

The raw data supporting the conclusions of this article will be made available by the authors upon request.
